# Ureteroscopy-assisted retrograde nephrostomy for lower calyx calculi in horseshoe kidney: two case reports

**DOI:** 10.1186/1752-1947-6-194

**Published:** 2012-07-10

**Authors:** Takashi Kawahara, Hiroki Ito, Hideyuki Terao, Katsuyuki Tanaka, Takehiko Ogawa, Hiroji Uemura, Yoshinobu Kubota, Junichi Matsuzaki

**Affiliations:** 1 Department of Urology, Ohguchi Higashi General Hospital, 2-19-1, Irie, Kanagawa-ku, Yokohama, Kanagawa, Japan; 2 Department of Urology, Yokohama City University, Graduate School of Medicine, 3-9, Fukuura, Kanazawa-ku, Yokohama, Kanagawa, Japan; 3 Department of Urology, Knagawa Rehabilitation Hospital, 713, Nanasawa, Atsugi, Kanagawa, JAPAN

**Keywords:** Ureteroscopy, Horseshoe kidney, Lawson catheter, Retrograde nephrostomy, PCNL

## Abstract

**Introduction:**

We previously reported on the effectiveness of ureteroscopy-assisted retrograde nephrostomy during percutaneous nephrolithotomy and report two cases of lower calyx calculi in horseshoe kidney that were successfully treated with ureteroscopy-assisted retrograde nephrostomy. During the ureteroscopy-assisted retrograde nephrostomy procedure, a ureteroscope is advanced in the desired calyx and a Lawson retrograde nephrostomy puncture wire is inserted. The wire is advanced through the calyx to exit the skin. The wire is then used for the percutaneous dilation.

**Case presentation:**

Case 1 was a 68-year-old man who was shown on radiography to have left lower calyx calculi (19 × 15mm, 7 × 5mm, and 7 × 3mm) in horseshoe kidney. Case 2 was a 36-year-old woman shown on radiography to have a left lower calyx calculus (10 × 8mm) in horseshoe kidney.

**Conclusions:**

Both patients were stone-free after ureteroscopy-assisted retrograde nephrostomy during percutaneous nephrolithotomy. Ureteroscopy-assisted retrograde nephrostomy is a promising procedure for safely and effectively treating lower calyx stones in horseshoe kidney.

## Introduction

Horseshoe kidney is the most common of all renal fusion anomalies, with a prevalence of 0.25% in the general population [1]. Some patients have been successfully treated with ureteroscopy, although due to the altered anatomical relationships in this disorder, ureteroscopic approaches can be quite challenging and are not universally recommended [2,3]. In several small case series, percutaneous nephrolithotomy (PCNL) has been shown to be highly successful, with an overall stone-free rate of 89% [4]. PCNL is, therefore, considered to be suitable for treating renal calculi in the lower calyx in horseshoe kidney. However, performing nephrostomy on the target calyx is difficult without dilating the renal collecting system even if an occlusion balloon catheter is used to create hydronephrosis.

We previously reported on the effectiveness of ureteroscopy-assisted retrograde nephrostomy (UARN) during PCNL [5]. With UARN we can approach the target puncture site easily under continuous visualization. We report two cases of lower calyx calculi in the horseshoe kidney successfully treated with UARN.

## Case presentation

### Case1

**Figure 1:**
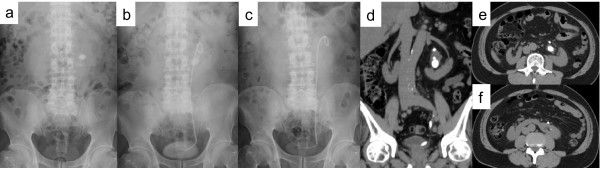
**Case 1: (a) preoperative kidneys, ureters, and bladder X-ray films, (b) preoperative stenting, and (c) postoperative.** Preoperative non-contrast (**d**) coronal and (**e**, **f**) axial computed tomography images.

A 68-year-old man referred to our hospital was shown on radiography to have left lower calyx calculi (19 × 15mm, 7 × 5mm, and 7 × 3mm) in horseshoe kidney (Figure 1a and b, d–f). He had no particular past medical history. His laboratory data showed no remarkable findings except for microhematuria on urinary analysis. In September 2011, he was admitted to our department for PCNL.

### Case2

**Figure 2:**
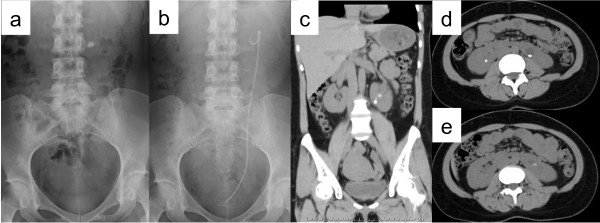
**Case 2: (a) preoperative and (b) postoperative kidneys, ureters, and bladder X-ray films.** Preoperative non-contrast (**c**) coronal and (**d**, **e**) axial computed tomography images.

A 36-year-old woman referred to us was found on radiography to have a left lower calyx calculus (10 × 8mm) in horseshoe kidney (Figure 2a, c–e). She had no remarkable previous or family history. Her laboratory data was also unremarkable. In July 2011, she was admitted to our department for PCNL.

### Procedure

**Figure 3:**
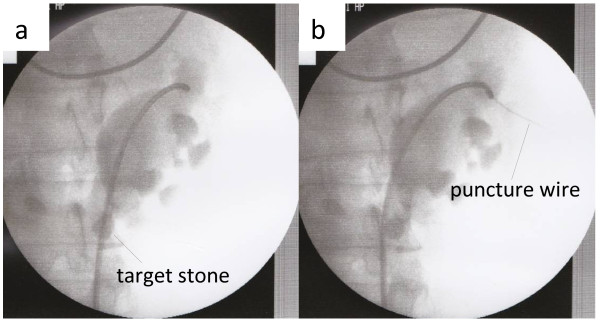
Retrograde pyelography in Case 1.

**Figure 4:**
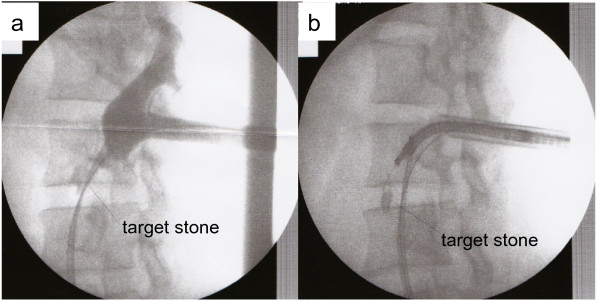
**Ureteroscopy-assisted retrograde nephrostomy procedure in Case 1.** (**a**) Inserting the puncture wire from the target calyx. (**b**–**e**) show catheter and balloon dilation. (**f**) Nephron access sheath insertion.

**Figure 5:**
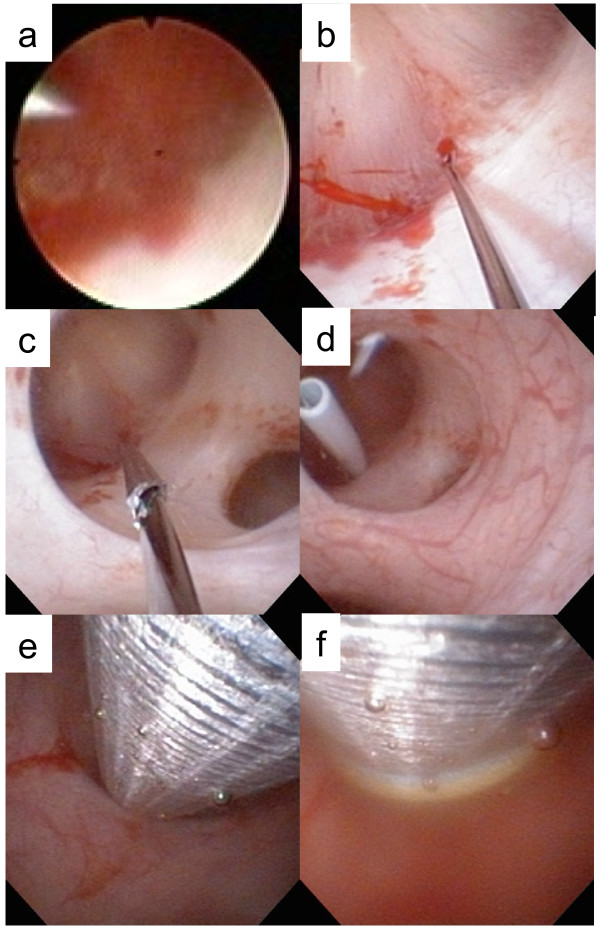
(a) Retrograde pyelography and (b) intraoperative image in Case 2.

**Figure 6:**
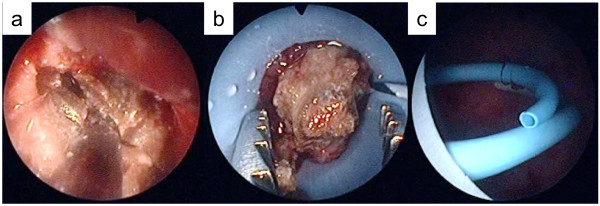
Percutaneous nephrolithotomy procedure in Case 1.

Under general and epidural anesthesia, the patient was placed in a modified-Valdivia position (Galdakao-modified Valdivia position) [6]. A flexible ureteroscope (URS) (Flex-X2™, Karl Storz, Germany) was inserted through a 13-French (inner diameter) ureteral access sheath (UAS) (Navigator® 13 French, 36cm, Boston Scientific) inserted into the ureter. We imaged the target calculi and defined the appropriate position to puncture. However, because the ureteroscope could not reach as far as the target stones in the lower calyx in either patient, we decided on a puncture site in the upper calyx of the renal collecting system. A Lawson retrograde nephrostomy puncture wire (COOK Urological, USA) was advanced into the flexible ureteroscope. The scope was advanced again to the calyx and the puncture wire forwarded along the route from the upper calyx to the exit skin. (Figures 3a3b, and 4a in Case 1, Figure 5a and b in Case 2) To avoid injury to the spleen, intestines, and pleural cavity, we performed preoperative computed tomography (CT) to confirm the anatomical image from the target calix to the skin and performed the puncture after ultrasonography. The puncture wire passed through the muscle easily and ‘tented’ the skin at the posterior axillary line. The skin was incised and the needle delivered. Next, the dilator was placed over the puncture wire, which was advanced through the skin, subcutaneous fat, abdominal wall musculature, and perinephric fat until it reached the renal parenchyma under the ureteroscope (Figure 4b–d). A 30-French percutaneous nephro access sheath (NAS; X-Force® Nephrostomy Balloon Dilation Catheter, BARD) was then passed over the balloon under continuous visualization with the URS (Figure 4e and f). After inserting the NAS into the renal collecting system, calculus fragmentation was undertaken using the Swiss LithoClast® pneumatic lithotripter (EMS, Switzerland) through a rigid nephroscope (percutaneous nephroscope, Karl Stortz) (Figure 6a–c). A postoperative kidneys, ureters, and bladder (KUB) film was taken and is shown in Figure 1c for Case 1 and Figure 2b for Case 2. Operation time was 157 minutes in Case 1 and 105 minutes in Case 2. There were no major or minor complications. Stone analysis in Case 1 revealed calcium oxalate and in Case 2 revealed calcium phosphate (52%) and calcium oxalate (48%).

## Discussion

Most horseshoe kidneys are fused by the formation of an isthmus between the lower poles of the left and right kidneys during development [6]. Shock wave lithotripsy (SWL), ureteroscopy, PCNL, and open surgery are used for the treatment of renal stones in horseshoe kidney [1,3,6,7]. Although ureteroscopic lithotripsy for renal calculi in horseshoe kidney has been reported, accessing the lower pole with a ureteroscope is sometimes difficult due to the anatomical need for higher insertion of the ureter into the renal collecting system [1,2]. The usefulness of PCNL for renal calculi in horseshoe kidney has been reported [1,4,8-10].

To reach to the lower calyx, nephrostomy is usually created in the upper calyx. In patients with horseshoe kidney, Raj *et al.* reported that 15 out of 24 patients (64%) required nephrostomy in the upper calyx and Al-Otabi *et al.* reported that nine out of 12 patients (75%) required it [1,4]. An upper pole nephrostomy tract allows for enhanced intrarenal access to the upper pole calyx, renal pelvis, lower pole calyx, ureteropelvic junction, and proximal ureter [1]. The disadvantage of nephrostomy in the upper calyx is that it sometimes involves longer distances from the skin to the lower calyx, meaning that the nephroscope cannot reach the target stone [1,7]. Raj *et al.* reported a 6% rate of pneumothorax complications with nephrostomy in the upper calyx [1]. Clearly, an accurate puncture location for the upper calyx is needed. We performed preoperative CT in all cases and performed ultrasonography before puncture to avoid injuring the surrounding organs. We punctured under fluoroscopic guidance to avoid injury and in more than 40 cases of UARN we have successfully avoided injury above the 12th rib using fluoroscopic guidance.

Our procedure may make percutaneous nephrostomy possible and easy to perform. A previous report described PCNL as a safe and established procedure, although severe complications requiring blood transfusion can sometimes occur [11]. At our institute, ureteroscopy was successful in treating a number of cases of renal calculi in the lower calyx in horseshoe kidney. In these cases, we usually positioned the patient in the Galdakao-modified Valdivia position and performed ureteroscopy initially to confirm whether the ureteroscope could reach the target calyx or not. When it did not reach, percutaneous puncture under ultrasonographic or fluoroscopic guidance could be performed, but in all cases UARN afforded an accurate puncture based on the ureteroscopic findings.

A UAS can facilitate ureteroscopy and retrieval of stone fragments while reducing the intrarenal pressure, improving irrigation flow, and decreasing operation time [12-14]. At our institute, we do not routinely insert a UAS but use it to aid the approach only when necessary. A previous study showed that ureteral stents are useful for passive dilation of the ureter in children [13], so the policy at our institute is to place a ureteral stent seven to ten days before ureteroscopy to insert UAS. In case 1, we performed preoperative stenting seven days before this procedure. But, as a result, this pre-stenting might not be necessary for this procedure.

In UARN, after the needle has exited through the skin, no further steps are required in the preparation for dilation [15,16]. UARN is a promising procedure allowing accurate puncture for the desired calyx, and is expected to contribute to creating optimal nephrostomy in the upper calyx for PCNL in horseshoe kidney.

## Conclusion

UARN for PCNL is a safe and effective procedure in treating lower calyx calculi in horseshoe kidney.

## Consent

Written informed consent was obtained from the patients for publication of this manuscript and any accompanying images. A copy of the written consent is available for review by the Editor-in-Chief of this journal.

## Abbreviations

KUB, Kidney, ureter, bladder; NAS, Nephron access sheath; PCNL, Percutaneous nephrolithotomy; UAS, Ureteral access sheath; UARN, Ureteroscopy-assisted retrograde nephrostomy.

## Competing interests

The authors declare that they have no competing interests.

## Authors’ contributions

TK, HI, YT and JM analyzed the patients’ data regarding operation procedure. TK, KT, TO, HU and YK wrote the manuscript. All authors read and approved the final manuscript.
